# Zinc deficiency is associated with gynecologic cancer recurrence

**DOI:** 10.3389/fonc.2022.1025060

**Published:** 2022-11-24

**Authors:** Kazuho Nakanishi, Masafumi Toyoshima, Go Ichikawa, Shunji Suzuki

**Affiliations:** ^1^ Department of Obstetrics and Gynecology, Nippon Medical School Chiba Hokusoh Hospital, Chiba, Japan; ^2^ Department of Obstetrics and Gynecology, Nippon Medical School, Tokyo, Japan

**Keywords:** zinc deficiency, gynecologic cancer, predictor of recurrence, zinc acetate hydrate, zinc deficiency recurrence

## Abstract

Zinc deficiency can cause various symptoms, including hair loss, anemia, and taste disorders. Recently, the association between cancer and zinc deficiency has received much attention with respect to its antioxidant properties. However, only a few studies have investigated the association between gynecologic cancers and zinc; to date, no studies have evaluated serum zinc status at the onset of gynecologic cancer or the relationship between zinc and cancer recurrence. The objectives of the present study were to determine whether serum zinc concentrations are associated with the development of gynecologic cancer, to clarify serum zinc dynamics between the onset and recurrence of gynecologic cancer, and to identify the associated factors. Accordingly, we retrospectively determined serum zinc concentrations before treatment in gynecologic patients with benign disease or cancer at the Nippon Medical School Chiba Hokusoh Hospital. We investigated anemia and hypoalbuminemia—the most common causes of zinc deficiency—as indicators of hyponutrition to determine the causal relationship of this deficiency with chemotherapy, radiation therapy, and recurrence, which may affect zinc concentration during cancer recurrence. The results indicated that there was no difference in zinc concentration between preoperative cancer patients and noncancer patients and that serum zinc concentrations were not associated with developing gynecologic cancers. However, patients with gynecologic cancer exhibited significantly lower serum zinc concentrations following treatment, and patients with recurrent cancer were 4.8 times more likely to develop zinc deficiency than those with nonrecurrent cancer. A serum zinc concentration of <61 μg/dL was an independent predictor of recurrence. Once zinc deficiency occurred, the recurrence rate of zinc deficiency reached as high as 69%. Overall, our study indicates that zinc deficiency is associated with recurrence in gynecological cancers and physicians should monitor zinc levels during disease management.

## Introduction

Zinc deficiency is common in developing countries, and it affects more than 2 billion individuals worldwide (1). Adequate but not excessive zinc intake benefits the general population, and dietary zinc intake may reduce the risk of gastrointestinal cancers, depression, and type 2 diabetes in adults. Zinc supplementation in adults reportedly improves depression, sperm quality, attentiveness, pregnancy rates, and diarrhea, reduces the risk of pneumonia in children, improves zinc deficiency, and promotes growth. Moreover, it ameliorates respiratory tract infections (including COVID-19) because of its antiviral, antioxidant, and anti-inflammatory effects (2).

Because the human body cannot store zinc, a deficiency can occur relatively rapidly, such as from an improper diet. Numerous epidemiological studies have demonstrated a relationship between dietary zinc content and cancer risk. The anticancer effects of zinc are mostly related to its antioxidant properties (3). Being a trace element, zinc is essential in various metabolic processes, including protein synthesis, immune response, and gene expression (4, 5). Therefore, zinc deficiency can cause several disorders in the human body (**Table 1**) (4, 6–11).

**Table 1:** Symptoms caused by zinc deficiency.

• Alopecia • Anemia • Decreased immune function, infection • Dermatitis • Diarrhea • Gastrointestinal dysfunction • Hepatosplenomegaly • Hypogonadism • Intractable bedsores • Memory loss, cognitive impairment • Stomatitis • Taste disorder

According to the Japan’s Practical Guideline for Zinc Deficiency 2018 published by the Japanese Society of Clinical Nutrition, approximately 15% of the Japanese population has inadequate zinc intake (12, 13). Several reports have linked digestive cancers to zinc, suggesting that its intake can reduce the risk of digestive, colorectal, and pancreatic cancers (14–17); however, few studies have demonstrated a relationship between gynecologic cancers and zinc intake. Only three previous observational studies have shown that compared with healthy adults, patients with ovarian, endometrial, and cervical cancers exhibit lower zinc concentrations (18–20).

A comprehensive PubMed search using the search terms “zinc” (MeSH term) and “gynecologic cancer” (MeSH term) revealed only one report describing changes in serum zinc concentrations before and after treatment of gynecologic cancer. Yanazume et al. prospectively evaluated 28 patients with suspected zinc deficiency before chemotherapy and concluded that chemotherapy may be a risk factor for low zinc concentrations (21). No studies have evaluated serum zinc status at the onset of gynecologic cancer (i.e., zinc deficiency associated with the development of gynecologic cancer) or the relationship of zinc to cancer recurrence. Therefore, the objectives of the present study were to determine whether serum zinc concentrations are associated with gynecologic cancer development, to identify factors that affect serum zinc concentration at the time of recurrence, and to clarify serum zinc dynamics and associated factors between gynecologic cancer development and recurrence. We measured serum zinc concentrations in gynecologic patients with benign disease or cancer treated at the Department of Obstetrics and Gynecology, Nippon Medical School, Chiba Hokusoh Hospital; we determined the relationship between serum zinc concentrations and the development, treatment, and recurrence of gynecologic cancers.

## Ethical approval

The purpose of the study was explained and written informed consent was obtained from all patients. In addition, approval was obtained from the Ethics Committee of the Nippon Medical School, Chiba Hokusoh Hospital (approval number: H-2022-003).

## Materials and methods

### Patient history

From 1/1/2019 to 12/31/2021, serum zinc concentrations of 214 patients with gynecologic cancer were measured at the Nippon Medical School Chiba Hokusoh Hospital, Chiba, Japan. As controls, 120 patients with benign gynecologic diseases before surgery were enrolled. Patients diagnosed with zinc deficiency were treated with oral zinc supplements at 30 mg per day for 30 days (zinc acetate hydrate, Nobelpharma Co., Ltd., Tokyo, Japan) and they received nutritional guidance including a high zinc diet.

### Study design

Serum zinc and blood hemoglobin (Hb) concentrations were measured before surgery, during chemotherapy, and at the follow-up examination after treatment in 214 patients with gynecologic cancer, which included 53 patients with cervical cancer, 90 with endometrial cancer, 67 with ovarian cancer, and four with other cancers. Patient body mass index (BMI), treatment history (total hysterectomy or bilateral adnexectomy, chemotherapy, and radiotherapy), recurrence status, and time from the previous treatment to recurrence were evaluated.

### Blood sample analysis

Blood samples were collected during preoperative testing, postoperative chemotherapy, and at follow-up visits after treatment completion. The samples were used to determine white blood cell, neutrophil, and lymphocyte counts using a multi-item automatic blood cell analyzer (XE-2100, Sysmex, Kobe, Japan). A chemistry autoanalyzer (LABOSPECT 008 α, Hitachi, Ibaraki, Japan) was used to measure serum albumin (Alb) levels based on the manufacturer’s instructions. Anemia was defined as a blood Hb level of <12 g/dL and hypoalbuminemia as a serum Alb level of <4.1 g/dL. A flame atomic absorption spectrophotometry method (SpectrAA-240, Agilent Technologies, Inc., California, USA) was used to measure serum zinc concentrations within the scope of the Japanese health insurance program using zinc standard solutions (Zn 1000, Cat. No. 48096-1B, 2B, Kanto Chemical Co., Inc., Saitama, Japan); this test was accredited by IA Japan, a member of the International Association for Laboratory Accreditation Cooperation and the Asia-Pacific Accreditation Cooperation for Mutual Recognition. The limit of detection for this test is 0.0009 μg cm^−3^ with a relative expanded uncertainty of 0.5% (coverage factor *κ* = 2; level of confidence, approximately 95%). A serum zinc concentration of <60 µg/dL was considered zinc deficiency. The timing of the serum zinc measurements was grouped into 6 periods: before treatment and 0–6, 7–12, 13–24, 25–36, and ≥37 months after treatment.

### Diagnosis of zinc deficiency

Zinc deficiency can be reliably diagnosed using the following three criteria (12, 13).

- One or more symptoms and signs of zinc deficiency including dermatitis, aphthous stomatitis, hair loss, loss of appetite, taste disorder, hypogonadism in males, anemia, increased infection susceptibility, growth disturbances in terms of weight and height in children, and low serum alkaline phosphatase (ALP) levels. However, serum ALP levels are not always low in patients with liver disease, osteoporosis, chronic kidney disease, or diabetes mellitus.
- Ruling out other diseases associated with the above symptoms or low serum ALP levels. For example, conditions including contact dermatitis, atopic dermatitis, dermatitis resulting from vitamin A, biotin, or essential fatty acid deficiencies, alopecia areata, hair-pulling, short stature resulting from growth hormone deficiency, familial short stature, Turner syndrome, and congenital hypophosphatasia should be ruled out.
- Low serum zinc concentrations
- 1. <60 µg/dL: zinc deficiency
- 2. 60–80 µg/dL: marginal zinc deficiency

Zinc supplementation can be provided to patients who meet criteria I, II, and III. Symptoms for these patients can be improved with zinc supplementation.

### Statistical analysis

Age and serum zinc concentration were analyzed using a t-test. Fisher’s exact test and logistic regression analysis were used to compare zinc deficiency to anemia, hypoalbuminemia, chemotherapy, radiation therapy, and recurrence. The Kruskal-Wallis rank sum test was used to compare serum zinc levels in patients with cervical, uterine, and ovarian cancer after treatment. Prediction of the therapeutic effects of serum zinc was determined using a receiver operating characteristic (ROC) curve. The effectiveness of the ROC curve was evaluated by the area under the curve (AUC) and the threshold was set at the point in which the sum of the sensitivity and specificity was maximized. The log-rank test was used to evaluate recurrence by serum zinc concentration. All statistical analyses were performed using EZR (Saitama Medical Center, Jichi Medical University, Saitama, Japan), which is a graphical user interface for R (The R Foundation for Statistical Computing, Vienna, Austria). It is a modified version of R commander, which is designed to add statistical functions frequently used in biostatistics (22).

## Results

As controls, 120 patients with benign gynecologic diseases before surgery were enrolled. For the comparison of cancer and noncancer patients, BMI was substituted as a measure of nutritional status because the blood Alb concentration of noncancer patients was only partially measured. The mean age ± standard deviation of cancer and noncancer patients was 61.1 ± 15.8 and 46.9 ± 13.0, respectively (p<0.001), so patients were matched for age with a caliper of 0.2 standard deviation (**Table 2**).

**Table 2:** Relationship between BMI, hemoglobin levels, and serum zinc concentrations in cancer and noncancer patients.

	Noncancer	Cancer	P-value
BMI (kg/m^2^)	23.5	24.2	0.452
Hemoglobin level (g/dL)	12.9	12.9	0.971
Zinc concentration (µg/dL)	69.4	71.9	0.259

BMI, Body mass index. *age was adjusted with a caliper of 0.2 standard deviation analysis of variance, and BMI was substituted as a measure of nutrition because blood albumin levels were not measured in all of the noncancer patients.

There were 214 patients with cancer in the analysis, including 53 patients with cervical cancer, 90 with endometrial cancer, 67 with ovarian cancer, and four with other cancers; their mean age was 58.8 years. Of these, 202 patients had undergone a total hysterectomy (types 1–3), 193 underwent bilateral adnexectomy, 117 were administered chemotherapy, 39 had undergone radiation therapy, and 35 experienced a recurrence. During the course of treatment or follow-up, serum zinc concentrations of <60 µg/dL were observed in 107 patients, and they were diagnosed with zinc deficiency (**Table 3**).

**Table 3:** Breakdown of patients with cancer included in the analysis.

N	214
Age (year ± SD)	58.8 ± 13.3
Cervical cancer (N)	53
Endometrial cancer (N)	90
Ovarian cancer (N)	67
Other cancer (N)	4
Total hysterectomy (N)	202
Bilateral adnexectomy (N)	193
Chemotherapy (N)	117
Radiation (N)	39
Recurrence (N)	35
No recurrence (N)	179
Zinc deficiency (N)	107

**Figure 1:**
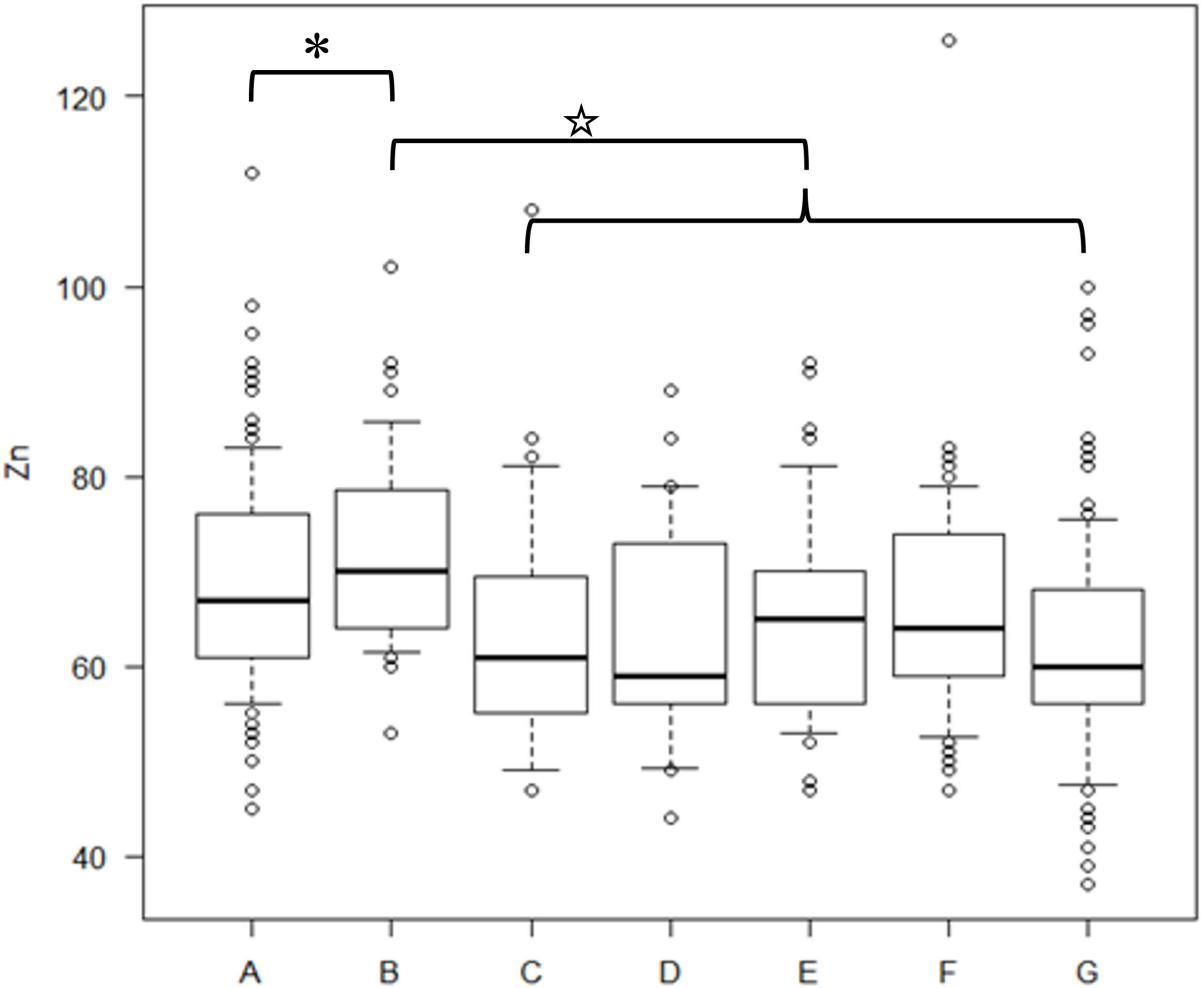
Serum zinc concentrations in cancer and non-cancer patients and cancer patients during the post-treatment period The concentration of serum zinc concentrations in each group. A) preoperative noncancer patients, B) preoperative cancer patients, C) within six months after treatment, D) 7–12 months after treatment, E) 13–24 months after treatment, F) 25–36 months after treatment, G) ≥37 months post-treatment. A t-test was used for the statistics of each group’s zinc concentrations. P-values for the difference in zinc concentrations between B and C, B and D, B and E, B and F, and B and G were 0.00812, 0.00689, 0.00104, 0.00905, and 0.0000109, respectively. * indicates no significant difference between A and B. ☆ indicates lower serum zinc concentrations in the C–G group than in B.

Preoperative serum zinc concentrations did not differ between noncancer (69.4 ± 11.1 μg/dL) and cancer (71.9 ± 10.3 μg/dL) patients (p = 0.259). Of the 214 patients, 35 patients with recurrent cancer (mean age, 58.4 years) and 179 with nonrecurrent cancer (mean age, 61.2 years) were observed. Following initial treatment, 107 patients (50%) were diagnosed with zinc deficiency. The mean time from cancer treatment to the onset of zinc deficiency was 30.8 months. The mean ± standard deviation of the lowest serum zinc concentration following treatment in the cervical, endometrial, and ovarian cancer groups was 59.8 ± 10.6, 59.4 ± 8.9, and 58.3 ± 11.4 μg/dL, respectively, with no significant difference among the three groups (Kruskal–Wallis rank sum test, p = 0.4485). Compared with pretreatment serum zinc concentrations, the concentrations at 6 months, 7–12 months, 13–24 months, 25–36 months, and ≥37 months post-treatment were significantly lower in patients with cancer (**Figure 1**). There were no differences in zinc concentrations between the groups after treatment.

Because we observed significant differences in the association of zinc deficiency after cancer treatment with anemia (Hb < 12 g/dL), hypoalbuminemia (Alb < 4.1 g/dL), chemotherapy, and radiotherapy (**Table 4**), we performed a logistical regression analysis on binary variables (**Table 5**). The results showed that only recurrence was significantly associated with zinc deficiency with an odds ratio of 4.8 (p = 0.0117). Of the 35 patients with recurrent cancer, 29 (83%) developed zinc deficiency. Of these, 27 developed zinc deficiency after recurrence, whereas 2 developed zinc deficiency before recurrence. The mean ± standard deviation time from recurrence to the onset of zinc deficiency was 16.9 ± 14.9 months, ranging from −5 to 63 months. The lowest serum zinc concentrations in patients with recurrent and nonrecurrent cancer were 54.0 ± 6.9 and 60.6 ± 10.1 μg/dL (p = 0.000378), respectively.

**Table 4:** Fisher’s exact test for each factor with and without zinc deficiency following cancer treatment.

	Anemia	Hypoalbuminemia		Radiation		Chemotherapy		Recurrence		
	(+)	(−)	(+)	(−)	(+)	(−)	(+)	(−)	(+)	(−)
Zinc deficiency (+)	30	77	26	81	23	84	67	40	30	77
Zinc deficiency (−)	9	62	9	62	10	61	32	39	4	67
p-value	0.0165†	0.082		0.242		0.0307†		0.000155†		

All were calculated with Fisher’s exact test. † means p-value of <0.05.

Anemia (Hb < 12g/dL), hypoalbuminemia (Alb < 4.1g/dL), and zinc deficiency (Zn < 60 μg/dL) according to institutional criteria.

**Table 5:** Logistic regression analysis on zinc deficiency following cancer treatment.

	**Odds ratio**	**95% CI**	**p-value**
Anemia	1.09	0.389–3.07	0.867
Hypoalbuminemia	1.42	0.556–3.65	0.462
Chemotherapy	1.35	0.683–2.68	0.386
Radiation	1.18	0.492–2.84	0.707
Recurrent	4.8	1.42–16.2	0.0117^†^

CI, confidence interval. All values were calculated with logistic regression analysis on binary variables. † means p-value of <0.05. Anemia (Hb < 12g/dL), hypoalbuminemia (Alb < 4.1g/dL), and zinc deficiency (Zn < 60 μg/dL) according to institutional criteria.

Next, we examined whether the decline in serum zinc concentrations in patients with recurrent cancer was influenced by the cancer treatment itself (chemotherapy or radiation therapy), weight changes because of cancer progression, or age. A comparison of 35 patients with recurrent and 179 with nonrecurrent cancer showed differences in BMI, chemotherapy, and radiotherapy (**Table 6**). We matched BMI with a caliper of 0.2 standard deviation, performed a matched-pair logistic regression analysis of chemotherapy and radiation therapy; we observed no difference in prior treatment between patients with recurrent and nonrecurrent cancer (**Table 7**). This indicates that zinc deficiency in patients with recurrent cancer is not caused by chemotherapy or radiotherapy but by the recurrence itself.

**Table 6:** Comparison of patients with recurrent and nonrecurrent cancer following cancer treatment.

	**Odds ratio**	**95% CI**	**p-value**
BMI	0.83	0.75–0.92	0.00043^†^
Age	1.02	0.98–1.06	0.3
Radiation	3.31	1.20–9.12	0.021^†^
Chemotherapy	13.00	3.57–47.10	0.000099^†^

BMI, Body mass index; CI, confidence interval. All values were calculated with logistic regression analysis on binary variables. † means p-value of <0.05

**Table 7:** Comparison of patients with recurrent and nonrecurrent cancer following cancer treatment (matched-pair logistic regression analysis with BMI matched by caliper of 0.2 standard deviations).

	**Odds ratio**	**95% CI**	**p-value**
Chemotherapy	1.26 × 10^8^	0.00–Inf	0.998
Radiation	3.00	0.61–14.90	0.178

BMI, Body mass index; CI, confidence interval. All were calculated by matched-pair logistic regression analysis with BMI matched by a caliper with a standard deviation of 0.2.

**Figure 2:**
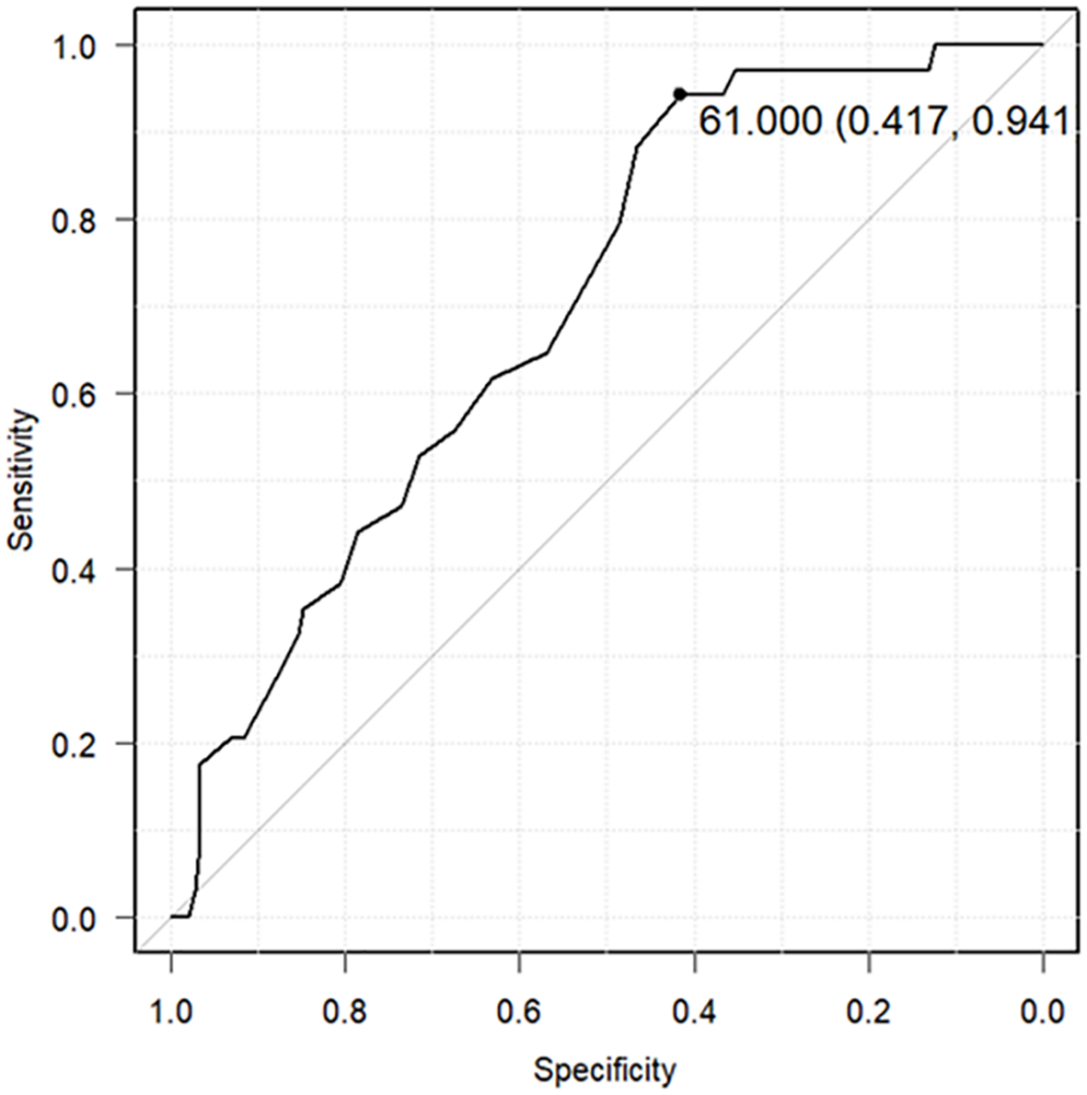
Receiver operating characteristic (ROC) curves predicting cancer recurrence using the lowest serum zinc concentrations. The ROC curve predicts cancer recurrence using a cutoff of 61 μg/dL as the lowest serum zinc concentration (sensitivity: 0.417, specificity: 0.941, area under the ROC curve: 0.698, 95% confidence interval: 0.611–0.785).

**Figure 3:**
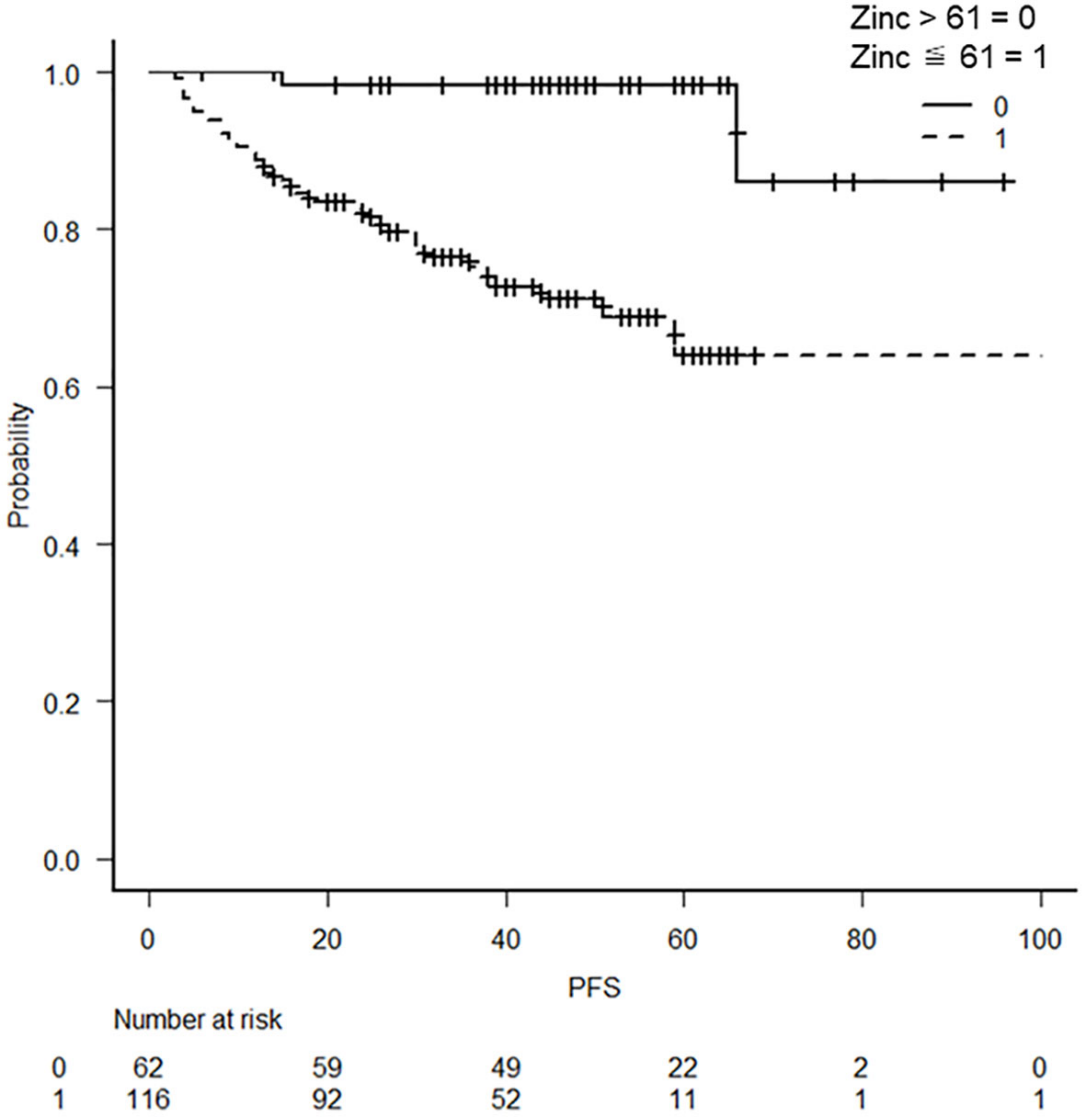
Kaplan–Meier curves with a cutoff at the lowest blood zinc concentration of 61 μg/dL Kaplan–Meier curves show a worse prognosis for serum zinc concentrations of ≤61 μg/dL (p = 0.0000244).

The relationship between the lowest serum zinc concentration and recurrence was evaluated using ROC curves. A cutoff serum zinc concentration of ≤61 μg/dL was associated with recurrence with a sensitivity of 0.417, a specificity of 0.941, and an AUC of 0.698 (95% confidence interval: 0.611–0.785) (**Figure 2**). The prognosis for recurrence-free survival was markedly worse in the group with a minimum zinc concentration of ≤61 μg/dL (p = 0.0000244) (**Figure 3**).

**Figure 4:**
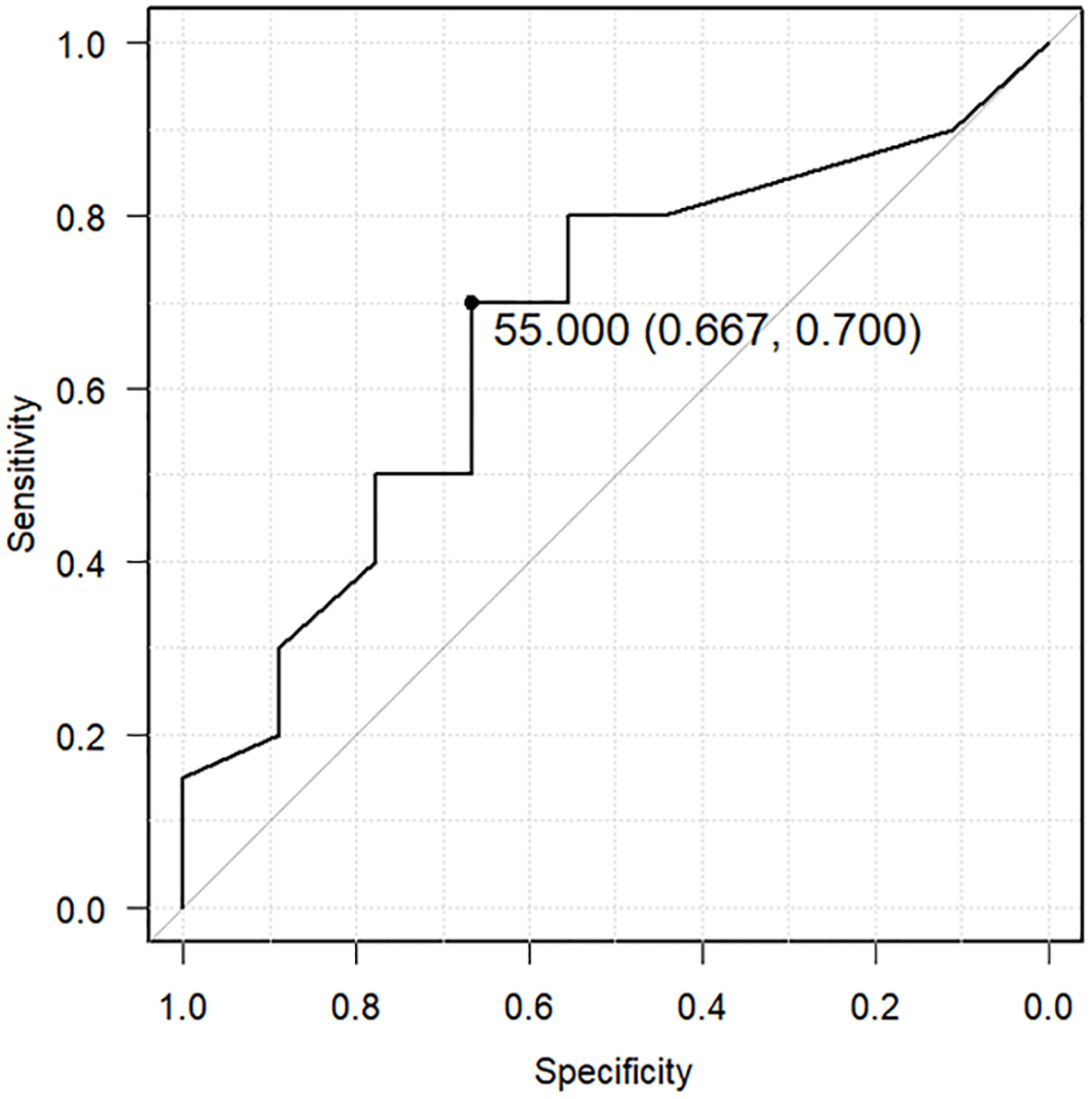
Receiver operating characteristic (ROC) curve predicting recurrent zinc deficiency using the lowest serum zinc concentration. The ROC curve predicts the recurrence of zinc deficiency using a cutoff of 55 μg/dL as the lowest serum zinc concentration (sensitivity: 0.667, specificity: 0.7, area under the ROC curve: 0.669, 95% confidence interval: 0.452–0.887).

All patients who developed zinc deficiency were treated with an oral zinc supplement and received additional nutritional guidance for a zinc-rich diet. Nevertheless, among patients with recurrent cancer who had developed zinc deficiency once, 20 of 29 (69%) developed zinc deficiency again. Therefore, we examined the recurrence of zinc deficiency in patients with recurrent cancer using the lowest serum zinc concentration as a predictor. A ROC curve predicted recurrence of zinc deficiency using a cutoff of 55 μg/dL as the lowest serum zinc concentration (sensitivity: 0.667, specificity: 0.7, area under the ROC curve: 0.669, 95% confidence interval: 0.452–0.887) (**Figure 4**).

## Discussion

In the present study, zinc concentrations decreased from pretreatment to post-treatment in patients with cancer. The development of zinc deficiency was significantly associated with recurrence, with 83% of patients with recurrent cancer developing zinc deficiency. We observed that serum zinc concentrations of ≤61 μg/dL were associated with cancer recurrence with an AUC of 0.698. Furthermore, a serum zinc concentration of ≤61 was associated with a worse prognosis compared with concentrations >61 μg/dL. The mean time from recurrence to the onset of zinc deficiency was approximately 17 months. Moreover, the risk of recurrent zinc deficiency was higher when the minimum serum zinc concentration was <55 μg/dL.

Concerning the relationship between zinc and gynecologic cancer, Lightman et al. (1986) first reported that zinc concentrations in ovarian tumor tissue were significantly lower compared with those in benign tissue specimens (23). Moreover, it was estimated that patients with cancer possess lower serum zinc concentrations than noncancer patients and that dietary zinc deficiency in terms of the antioxidant properties of zinc may result in DNA damage through oxidative modification, thereby increasing cancer risk (24). Previous studies have hypothesized that a decrease in serum zinc concentration resulting from diet or other factors triggers a reduced in its concentration in tumor tissue. This reduces the antioxidant effects of serum zinc and leads to the oxidative modification of DNA, thereby resulting in carcinogenesis.

Nonetheless, in the present study, the cancer patient group maintained similar preoperative zinc concentrations compared with the noncancer patient group. In addition, 69% of patients with recurrent cancer, who once developed zinc deficiency, developed zinc deficiency again, despite being treated with oral zinc supplements until their serum zinc concentrations improved and being provided nutritional guidance to adopt a zinc-rich diet. Therefore, in our cohort of patients with cancer, a decrease in zinc concentration because of an inappropriate diet did not contribute to carcinogenesis and recurrence was associated with zinc deficiency development.

Because our results showed a decrease in serum zinc concentration following cancer treatment, we predicted that factors associated with post-treatment nutritional status as well as other factors may have contributed to reducing serum zinc concentrations. Therefore, we considered chemotherapy and radiotherapy, which result in decreased dietary intake, as possible factors associated with zinc deficiency; moreover, factors such as anemia (25) and hypoalbuminemia (26), which are indicators of malnutrition and are correlated with zinc concentrations, were considered. However, we observed that only cancer recurrence was associated with zinc deficiency.

Because 87% of patients with recurrent cancer experienced zinc deficiency, it is possible that some of the symptoms associated with this condition, such as taste disorders and anemia, which also frequently occur in these patients, can be attributed to zinc deficiency (**Table 1**). However, our study did not retrospectively examine symptoms caused by zinc deficiency; hence, it is unclear the manner in which it affects the quality of life of patients with recurrent cancer. Therefore, further studies are required.

We observed that low serum zinc concentrations were independently associated with cancer recurrence after adjusting for age, anemia, hypoalbuminemia, and prior cancer therapy. Because zinc deficiency is associated with poor prognosis, measurement of serum zinc concentrations may be incorporated into treatment protocols for gynecologic cancers; however, further studies are required to determine whether zinc supplementation improves patient prognosis. Although the analysis of clinical symptoms associated with zinc deficiency in patients was not performed in the present study, patients with zinc deficiency who complained of alopecia or taste disorders not related to chemotherapy were rare. In contrast to some studies suggesting that low zinc concentrations trigger carcinogenesis, the occurrence of zinc deficiency occurred mostly after cancer recurrence, with a mean time between recurrence and occurrence of zinc deficiency of 17 months.

In patients with recurrent disease who exhibited zinc deficiency once, 20 of 29 (69%) developed zinc deficiency again. Moreover, zinc deficiency was likely to recur when the lowest serum zinc concentration fell below 55 μg/dL. Chemotherapy and radiation therapy as well as a low nutritional status of patients were not associated with zinc deficiency, leading to our hypothesis that cancer recurrence induces zinc deficiency. Furthermore, if quality of life is affected by zinc deficiency, perhaps oncologists should be more proactive in measuring post-treatment zinc concentrations and treating zinc deficiency. In particular, patients with serum zinc concentrations of <55 μg/dL should be carefully monitored.

One limitation of our study is that we did not monitor the nutritional intake of patients with recurrent cancer; hence, we could not analyze whether reduced dietary intake resulting from cancer recurrence was associated with zinc deficiency. However, because 69% of the patients with zinc deficiency who received appropriate oral zinc supplements and nutritional guidance for a high zinc diet experienced recurrent zinc deficiency, it may be inferred that in patients with recurrent cancer, other causes of zinc deficiency besides diet can be involved. Because our study was a single-center, retrospective study, the impact of zinc deficiency on the quality of life of patients with cancer remains unknown and prospective studies at multiple centers are warranted.

## Conclusions

No differences in serum zinc concentrations between patients with benign gynecologic disease and those with gynecologic cancer were observed before treatment. Patients with gynecologic cancer are at higher risk for developing zinc deficiency after initial treatment completion, and patients with recurrent cancer are at higher risk of zinc deficiency. Low serum zinc concentration is an independent factor associated with cancer recurrence and poor prognosis. Furthermore, once zinc deficiency develops, the risk of zinc deficiency recurrence is high. Therefore, gynecologic oncologists should actively measure serum zinc concentrations to improve prognosis and maintain quality of life in patients with recurrent cancer.

## Data availability statement

The raw data supporting the conclusions of this article will be made available by the authors, without undue reservation.

## Ethics statement

The studies involving human participants were reviewed and approved by Nippon Medical School Chiba Hokusoh Hospital Ethics Committee, Nippon Medical School Chiba Hokusoh Hospital. The patients/participants provided their written informed consent to participate in this study.

## Author contributions

MT and GI contributed to the conception and design of the study. KN organized the database, performed experiments, and wrote the manuscript. SS approved the final draft of the article. All authors contributed to the article and approved the submitted version.

## Acknowledgments

The authors would like to thank Enago (www.enago.jp) for the English language review.

## Conflict of interest

The authors declare that the research was conducted in the absence of any commercial or financial relationships that could be construed as a potential conflict of interest.

## Publisher’s note

All claims expressed in this article are solely those of the authors and do not necessarily represent those of their affiliated organizations, or those of the publisher, the editors and the reviewers. Any product that may be evaluated in this article, or claim that may be made by its manufacturer, is not guaranteed or endorsed by the publisher.
